# Aerobic Exercise Training-Induced Changes on DNA Methylation in Mild Cognitively Impaired Elderly African Americans: Gene, Exercise, and Memory Study - GEMS-I

**DOI:** 10.3389/fnmol.2021.752403

**Published:** 2022-01-17

**Authors:** Julius S. Ngwa, Evaristus Nwulia, Oyonumo Ntekim, Fikru B. Bedada, Bernard Kwabi-Addo, Sheeba Nadarajah, Steven Johnson, William M. Southerland, John Kwagyan, Thomas O. Obisesan

**Affiliations:** ^1^Division of Cardiovascular Medicine, Department of Internal Medicine, Howard University, Washington, DC, United States; ^2^Department of Psychiatry and Behavioral Sciences, Howard University, Washington, DC, United States; ^3^Department of Nutritional Sciences, Howard University, Washington, DC, United States; ^4^Department of Clinical Laboratory Sciences, Howard University, Washington, DC, United States; ^5^Department of Biochemistry and Molecular Biology, Howard University, Washington, DC, United States; ^6^Division of Nursing, Howard University, Washington, DC, United States; ^7^School of Nursing and Allied Health Sciences, Howard University, Washington, DC, United States; ^8^Department of Medicine, Howard University, Washington, DC, United States; ^9^Division of Geriatrics, Department of Medicine and Clinical/Translational Science Program, Howard University Hospital, Washington, DC, United States; ^10^Georgetown-Howard U Center for Clinical and Translation Science (GHUCCTS), Howard University Hospital, Washington, DC, United States

**Keywords:** African Americans, Alzheimer’s disease, CPG Islands, DNA methylation, mild cognitive impairment, VO_2_max

## Abstract

**Background:**

DNA methylation at CpG sites is a vital epigenetic modification of the human genome affecting gene expression, and potentially, health outcomes. However, evidence is just budding on the effects of aerobic exercise-induced adaptation on DNA methylation in older mild cognitively impaired (MCI) elderly African American (AAs). Therefore, we examined the effects of a 6-month aerobic exercise-intervention on genome-wide DNA methylation in elderly AA MCI volunteers.

**Design:**

Elderly AA volunteers confirmed MCI assigned into a 6-month program of aerobic exercise (eleven participants) underwent a 40-min supervised-training 3-times/week and controls (eight participants) performed stretch training. Participants had maximal oxygen consumption (VO_2_max) test and Genome-wide methylation levels at CpG sites using the Infinium HumanMethylation450 BeadChip assay at baseline and after a 6-month exercise program. We computed false discovery rates (FDR) using Sidak to account for multiplicity of tests and performed quantitative real-time polymerase chain-reaction (qRT-PCR) to confirm the effects of DNA methylations on expression levels of the top 5 genes among the aerobic participants. CpG sites identified from aerobic-exercise participants were similarly analyzed by the stretch group to quantify the effects of exercise-induced methylation changes among the group of stretch participants.

**Results:**

Eleven MCI participants (aerobic: 73% females; mean age 72.3 ± 6.6 years) and eight MCI participants (stretch: 75% female; mean age 70.6 ± 6.7 years) completed the training. Aerobic exercise-training was associated with increases in VO_2_max and with global hypo- and hypermethylation changes. The most notable finding was CpG hypomethylation within the body of the *VPS52* gene (*P* = 5.4 × 10^–26^), a Golgi-associated protein, involved in intracellular protein trafficking including amyloid precursor protein. qRT-PCR confirmed a nearly twofold increased expression of *VPS52*. Other top findings with FDR *q*-value < 10^–5^, include hypomethylations of *SCARB1* (8.8 × 10^–25^), *ARTN* (6.1 × 10^–25^), *NR1H2* (2.1 × 10^–18^) and *PPP2R5D* (9.8 × 10^–18^).

**Conclusion:**

We conclude that genome-wide DNA methylation patterns is associated with exercise training-induced methylation changes. Identification of methylation changes around genes previously shown to interact with amyloid biology, intracellular protein trafficking, and lipoprotein regulations provide further support to the likely protective effect of exercise in MCI. Future studies in larger samples are needed to confirm our findings.

## Background

Epigenetic mechanisms and their effects on gene activation and silencing are becoming increasingly relevant to phenotype expression and the development of different diseases (Bird, 2007; Gluckman et al., 2009). The epigenome, along with the genome, instructs the unique gene expression program of each cell type in order to define its functional identity during development or disease (Rivera and Ren, 2013). While epigenetic changes are functionally relevant to the genome, they do not involve changes in a DNA sequence. One of such epigenetic modifications is the methylation of cytosine molecule, usually at CG dinucleotides (CpGs), called DNA methylation. Thus, the addition of methyl groups to the DNA molecules, motivate changes in the DNA segment activity without changing its sequence. When the CpGs of promoter regions are methylated, gene expression are often silenced (Hashimshony et al., 2003). Specifically, DNA methylation may affect the transcription of genes in two ways: (a) physically impede the binding of transcriptional proteins to the gene; (b) may be bound by proteins known as methyl-CpG-binding domain proteins resulting in compact, inactive chromatin (heterochromatin) (Choy et al., 2010). There is a growing understanding that environmental manipulation (e.g., diet and exercise) can influence cell behavior and disease states through epigenetic alterations of gene expression.

Exercise is a well-known physiological stimulus resulting in health and functional improvements. Regular exercise has numerous health benefits and can help reduce the risk of common ailments such as cardiovascular disease, type II diabetes, several forms of cancer (Matheson et al., 2013), and importantly, neurodegeneration and cognitive deterioration. Until recently, there has been paucity of knowledge on how aerobic exercise can induce epigenetic modifications in humans. A global study of DNA methylation in human skeletal muscle from relatives of Type 2 Diabetes patients demonstrated that a 6-month exercise resulted in epigenetic changes (Nitert et al., 2012). However, whether aerobic exercise can influence DNA methylations in elderly AA MCI participants has not been examined. It is also unknown whether such changes will include genes having essential roles in neurodegeneration and cognitive decline.

Therefore, we examined the impact of a 6-month aerobic exercise-training on human DNA methylation in mild cognitively impaired (MCI) elderly African Americans (AA)s; identify DNA methylated genes affected by aerobic exercise, and investigated the biological pathways affected by aerobic exercise training-related changes in CpG intensities. We then used quantitative real-time polymerase chain reaction (qRT-PCR) to confirm the effects of these methylation changes on the mRNA levels of the associated genes.

## Materials and Methods

The Howard University Institutional Review Board (IRB) approved the protocols used for this investigation. As required for studies involving human subjects, all participants completed a signed informed consent form before enrollment in the study. The details of the Gene, Exercise, and Memory Study (GEMS-I) protocol have previously been published (Iyalomhe et al., 2015).

### Screening

Eligibility criteria consisted of age ≥ 55 years, ability to exercise vigorously without difficulty, have no chronic medical condition, met Petersen MCI criteria (Petersen et al., 1997) (age and education adjusted Score 24–30 inclusive), have memory complaints and objective memory loss (Mungas et al., 1996). Demographic and general medical history were obtained from volunteers after completing informed consent (Iyalomhe et al., 2015). Randomization of subjects to the intervention (aerobic participants) and control (stretch participants) groups occurred before baseline tests. All staff, except those directly monitoring exercise-training, were blinded to group assignments. The data were de-identified using assigned unique identifiers for labeling and tracking.

### Baseline Testing

Qualified participants underwent a maximal treadmill exercise test using the Bruce protocol (Bruce and Hornsten, 1969). Before randomization and baseline testing, participants maintained regular caloric intake and were instructed to continue throughout the study period. Except for those directly administering exercise-training, staff was blinded to group assignments. Baseline VO_2_max and endurance capacity were obtained using a modified Bruce protocol (Chaitman, 2001). Participants were instructed to abstain from alcohol, smoking, and anti-inflammatory medications 24 h before the blood draw. Fasting blood samples were obtained using sterile techniques and stored in heparinized collection tubes to enable gene expression analyses.

### Aerobic Exercise-Training Protocol

Individual maximal heart rate was inferred for both the intervention (aerobic exercise) and control (stretch exercise) groups from baseline VO_2_max tests before undergoing supervised training 3 times/week using the American College of Sports Medicine Guidelines (ACSM) (Pollock and Froelicher, 1990). Aerobic participants performed exercise training included a warm-up period followed by treadmill walking or jogging, stair-stepping, and elliptical, and an appropriate cool-down period. Initial training sessions lasted 20 min at 50% VO_2_max while monitoring protocol adherence using individual exercise heart rate and duration. Training duration increased by 5 min/week to 40 min at 50% VO_2_max, and then, incrementally by 5% VO_2_max/week until 70% VO_2_max was achieved. Additionally, participants underwent unsupervised 45–60 min lower intensity walk on weekends after the initial 4–6 weeks’ training.

### Stretch Training Protocol

Training of the stretch group consisted of maintaining exercise positions for 15–30 s to produce a slight pull on the muscle but not to the point of triggering the sensation of pain. Using different positions for a total of about 40 min, each stretch was directed at often tight muscles (e.g., hamstrings, hip flexors, calves, and chest) and repeated slowly, 3–5 times on each body side 3 days/week (Pollock et al., 1998).

### Follow-Up Test at 6 Months

After subjects completed the 6-month aerobic exercise or stretch training protocol, all baseline tests (VO_2_max, blood tests) were repeated.

### Sample Processing and Assessment of Methylation

Total DNA was isolated from clotted blood samples using a clotspin basket (Qiagen, Germany) to disperse the clot and then extracted using the MasterPure Complete DNA and RNA Purification Kit (Epicenter, cat#MC85200) according to the manufacturer’s instructions. DNA concentration and purity (OD260/280) were measured using NanoDrop ND-1000 spectrophotometer (Thermo Fischer Scientific, United States). A minimum of 500 ng DNA was used for bisulfite conversion using the EZ DNA Methylation kit (Zymo Research, United States) and a GeneAmp PCR system 9,700 (Applied Biosystems, United States), and cleaned up per manufacturer’s instructions. To confirm successful bisulfite modification, we subjected the DNA to PCR using methylation-specific primers. Methylation analysis was performed using the Illumina Infinium HumanMethylation450 Beadchip platform (Illumina Inc., United States). Briefly, bisulfite-modified DNA was fragmented into 300–600 bp fragments, purified by isopropanol precipitation, and resuspended in a hybridization buffer. The sample was then hybridized to an Illumina Infinium HumanMethylation450 Beadchip. The BeadChips were subsequently washed, stained, and dried according to the manufacturer’s instructions. The BeadChips were then scanned using a HiScanSQ System (Illumina Inc., United States). Methylation data were processed through Illumina GenomeStudio (Illumina Inc., United States) and analyzed in Partek (Partek Inc., St. Louis, MO, United States). Included in the methylation analysis are those who completed 6 months of intervention with methylation data.

### Pathway Analysis of CG Dinucleotide Sites in Aerobic Participants

We investigated the biological relevance of the genome-wide CpG sites associated with VO_2_max by considering the 248 CpG sites and performing Ingenuity Pathway Analysis on the gene annotated sites. Among these 248 CpG sites, 165 annotated to the human reference genome build 37 (hg19). We performed a Gene Ontology (GO) enrichment analysis for the genes encompassing or adjacent to these differentially methylated CpG sites.

### Quantitative Real-Time Polymerase Chain-Reaction

For quantitative RT-PCR analysis, total RNA was isolated from clotted blood samples using Trizol reagent according to the manufacturer’s instructions (Thermo Fisher Scientific, MA, United States). In the reverse transcription (RT) step, cDNA was reverse transcribed from 300 ng total RNA samples in 20 μl reaction buffer using the High-Capacity RT-kit (Thermo Fisher Scientific, MA, United States). Gene expression level was assessed through TaqMan expression assay system using standard 2x master mixes and 20x FAM-MGM labeled probe sets. The 20x FAM-MGM labeled probe sets used in the study have the following Assay IDs: Hs00987064_m1 for VPS52, Hs01027208_m1 for NR1H2, Hs00605059_m1 for PPP2R5D, and Hs00969821_m1 for SCARB1 and Hs99999905_m1 for GAPDH (Thermo Fisher Scientific, MA, United States). All qPCR assays were performed in duplicate samples and normalized against GAPDH and baseline control. Relative quantitation analysis of gene expression was conducted according to the 2^(–ΔΔCT^) relative expression method as described (Livak and Schmittgen, 2001; Bedada et al., 2014). GAPDH was used as an endogenous internal standard for expression analysis to determine the abundance of amplified target genes within the same sample. Reactions were monitored on Applied Biosystems ViiA™ 7 Real-Time PCR System and analyzed data with corresponding ViiA 7 RUO software.

## Statistical Analysis

Genome-wide methylation levels at CpG sites from participants at baseline and at 6 months after aerobic and stretch training were profiled using the Infinium HumanMethylation450 BeadChip assay (Illumina, San Diego, CA, United States). The raw intensity data of all samples were imported into the software Partek (St. Louis, MO, United States). SWAN method (Maksimovic et al., 2012) was selected to normalize array intensities. SWAN-normalized β values, which correspond to the percentage of methylation at a CpG site, were then calculated. Premised on the exclusion of probes with a detection *p*-value > 0.01 in one or more of the 22 samples for the downstream analysis, we discarded 6,942 probes while 478,570 probes remained.

Analyses examining baseline genome-wide CpG methylation and the influence of a 6-month aerobic exercise-training on methylations were conducted in STATA 14 software (StataCorp, 2015). First, all variables measured at baseline and post-6 months of exercise were examined descriptively, and their distributions were further inspected using Boxplots and Scatterplots (McGill et al., 1978; Royston and Cox, 2005). Student’s *t*-test was used to compute mean estimates of continuous variables between groups of baseline measures; nonparametric Mann–Whitney rank test to derive *p*-values for group differences in non-normal continuous variables; and Chi-square and Exact tests to compare group differences in categorical variables. For continuous measures, and within each group (aerobic and stretch), a paired *t*-test was used to assess significant differences in means between baseline and six months.

Effects of exercise-induced changes in VO_2_max (i.e., VO_2_max at baseline and after a 6-month training) on genome-wide CpG methylation were examined within-individual level using linear mixed-effects analysis (West et al., 2014), and robust variance-covariance approach adjusted for standard errors of the coefficients. This is robust to differences in variances between groups and to violation of normality assumption. Because sampling distributions of test statistics are known to be t and F-distributed in simple cases in small sample studies, we also used Satterthwaite and Kenward-Roger denominator-degrees-of-freedom (DDF) adjustments for small-sample inference. Given the sample size, we report standard errors derived from Kenward-Roger DDF more closely similar to the bootstrap estimation of standard errors of randomly selected CpG methylations (Satterthwaite, 1946; Kenward and Roger, 1997).

To account for the multiplicity of tests from the genome-wide inquiry, we used the frequentist *q*–values approach. The *q*-value package inputs a variable of *p*–values and outputs a variable of *q*-values, equal in each observation to the minimum FWER or FDR that would result in the inclusion of the corresponding *p*-value in the discovery set if the specified multiple–test procedure was applied to the complete set of input *p*–values (Newson, 2010). We computed false discovery rates (FDR) using Sidak approaches (Šidák, 1967).

## Results

### Characteristics of Aerobic and Stretch Participants

The baseline demographic characteristics (Table 1) were limited to aerobic participants (*n* = 11), and stretch participants (*n* = 8) who completed aerobic exercise- and stretch training had data for VO_2_max and methylation data. Continuous measures were summarized using means and proportions for categories. The aerobic sample consisted of 72.7% females (mean age of 71.3 ± 6.6 years) and BMI (mean 26.8 ± 4.5 kg/m^2^) at baseline. As anticipated, the participants had significant decreases in mean body weight (*p* = 0.007) after a 6-month training (158.8 ± 37.9 lbs.) compared to baseline (165.1 ± 6.0 lbs.). Although participants had increases in mean relative VO_2_max after 6 months (25.1 ± 7.8) compared to baseline (22.7 ± 2.7), the differences were not statistically significant (*p* = 0.414). The stretch sample consisted of 75.0% females (mean age of 70.58 ± 6.7 years) and BMI (mean 32.3 ± 5.5 kg/m^2^) at baseline. After 6 months of training, mean body weight remained relatively unchanged (*p* = 0.871) in the stretch group.

**TABLE 1:** Baseline characteristics of participants (completers with methylation data).

Characteristics	Aerobic (*N* = 11)			Stretch (*N* = 8)		
	Baseline	6 Months	*P*-value	Baseline	6 Months	*P*-value
Age–years.	72.35 (6.63)		–	70.58 (6.69)		–
Gender (Female%)	8 (72.73%)		–	6 (75.00%)		–
Weight (lb.)	165.09 (38.56)	158.82 (37.94)	0.007	190.63 (20.19)	189.00 (18.69)	0.871
BMI (kg/m^2^)	26.77 (4.55)	25.33 (3.69)	0.075	32.34 (5.47)	30.74 (3.33)	0.913
Max VO_2_ (Relative)	22.74 (2.69)	25.08 (7.82)	0.414	24.72 (8.68)	22.84 (3.93)	0.442
Max VO_2_ (Absolute)	1550.26 (532.42)	1870.75 (856.47)	0.252	2109.00 (597.85)	1934.00 (193.10)	0.411
Max systolic BP (mmHg)	169.89 (9.80)	–	–	200.00 (0.00)	–	–
Max diastolic BP (mmHg)	90.11 (11.94)	–	–	96.50 (9.19)	–	–
Max heart rate	147.91 (6.36)	146.80 (6.97)	0.003	149.63 (6.46)	145.43 (13.04)	0.302

*Values are mean ± SD when appropriate. P-values were computed with paired t-tests.*

### Genome-Wide DNA Methylation of CG Dinucleotide Sites in Aerobic Participants

**FIGURE 1:**
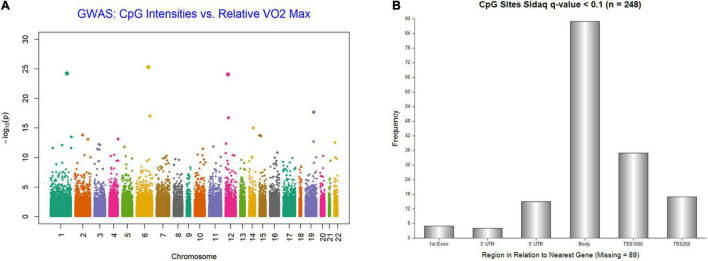
**(A)** Genome-wide plot for DNA methylation analysis of CpG intensities and relative VO_2_Max; distribution is based on location and chromosomal position for all 478,570 probes in Infinium HumanMethylation450 BeadChip assay. **(B)** Frequency of 248 CpG Islands by region to nearest Gene (sidakq < 0.1) that exhibited differential DNA methylation after 6-month aerobic exercise-training; 89 located in the Body; 35 in TSS1500; 17 in TSS200; 15 in 5′ UTR; 5 in 1st Exon; and 4 in the 3′ UTR regions to the nearest gene.

**FIGURE 2:**
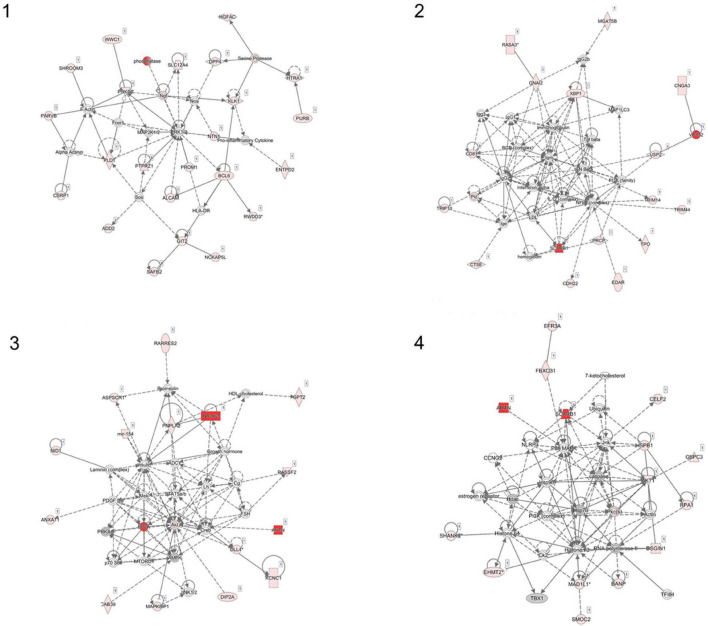
Ingenuity pathway network analysis (IPA) using top 248 CpG sites (network 1–4). Four networks identified with Ingenuity Pathway Analysis. **(1)** Cell morphology, cellular assembly, and organization, nervous system development and function; **(2)** Antimicrobial response, humoral immune response, infectious disease; **(3)** Carbohydrate metabolism, molecular transport, small molecule biochemistry; **(4)** Cancer, cell death and survival, DNA replication, recombination and repair.

We examined the association between the intensity changes in 478,570 individual CpG sites and VO_2_max after 6 months of aerobic exercise-training with adjustment for Age. As illustrated in genome-wide DNA methylation analysis of CpG intensities in Figure 1A, the distribution is based on the location and chromosomal position for all 478,570 probes in the Infinium HumanMethylation450 BeadChip assay. With the overall genomic inflation factor of all CpG sites being 1.03, the CpG sites exhibiting the most significant association with VO_2_max was cg00160018 (beta = −0.002, se = 0.0002, *p* = 5.38e-26), located in the body of the nearest gene *VPS52* (Table 2). Several other CpG sites nearest to *SCARB1*, *ARTN*, *NR1H2*, *PPP2R5D* genes exhibited significant associations (*p* < 10e-15). After applying FDR correction (Sidak *q*-value < 0.1), we identified 248 CpG sites that exhibited differential DNA methylation after the 6-month aerobic exercise-training. Among these 248 CpG sites, 89 were located in the body; 35 in TSS1500; 17 in TSS200; 15 in 5′ UTR; 5 in 1st Exon; and 4 in the 3′ UTR regions to the nearest gene (Figure 1B). A total of 214 CpG sites had a decrease in intensities after exercise compared to 34 CpG sites showing an increase. Pathway analysis of the top 248 CpG sites identified several biological pathways, including the top 4 biological networks depicted in Figure 2. To determine whether the top 10 CpG sites identified in the aerobic-exercise group were similarly influenced by stretch exercise, we performed a linear mixed-effects comparative analysis to quantify the effects of exercise-induced methylation changes in the stretch participants. However, the stretch group failed to demonstrate significant training-related changes for the top 10 CpG sites observed in the aerobic group, providing additional evidence that the observed changes in the aerobic exercise group was motivated by fitness adaptation (Supplementary Table 1).

**TABLE 2:** A 6-Month Aerobic Exercise Training–Induced Changes in DNA Methylation in African American MCI Subjects – Top 20 CpG Sites.

CpG	CHR	Gene Symbol	Gene Region	Percent	beta	se	*p*	sidakq
cg00160018	6	VPS52	Body	–0.71	–0.0020	0.0002	5.38E-26	0.00
cg11198639	12	SCARB1	Body	–2.55	–0.0063	0.0006	8.83E-25	0.00
cg17414508	1	ARTN	TSS1500	–1.23	–0.0049	0.0005	6.10E-25	0.00
cg24440997	19	NR1H2	TSS1500	–3.89	–0.0029	0.0003	2.06E-18	0.00
cg14385961	6	PPP2R5D	TSS200	–0.95	–0.0007	0.0001	9.83E-18	0.00
cg11132661	12			–0.92	–0.0018	0.0002	2.01E-17	0.00
cg02170785	14			–2.09	–0.0044	0.0006	9.71E-16	4.85E-10
cg02469461	2	CAB39	5′UTR	–0.48	–0.0015	0.0002	1.42E-14	6.90E-09
cg22988430	15	SNORD115-41	TSS200	–1.95	–0.0052	0.0007	1.84E-14	8.95E-09
cg08732418	15	DLL4	Body	–3.73	–0.0061	0.0008	2.13E-14	1.03E-08
cg14166197	1	CTNNBIP1	5′UTR	–1.11	–0.0027	0.0004	2.96E-14	1.44E-08
cg07794230	4			–0.66	–0.0018	0.0002	6.90E-14	3.35E-08
cg11124652	2			–2.05	–0.0064	0.0009	7.76E-14	3.77E-08
cg12104982	19	SAFB2	Body	–1.83	–0.0051	0.0007	1.95E-13	9.48E-08
cg15146966	22	XBP1	TSS1500	–1.78	–0.0011	0.0001	2.91E-13	1.41E-07
cg18926450	12	GIT2	3′UTR	–0.94	–0.0022	0.0003	4.69E-13	2.28E-07
cg22314684	3	PLD1	5′UTR	–1.39	–0.0033	0.0005	5.48E-13	2.66E-07
cg07403865	3	BCL6	5′UTR	–4.51	–0.0035	0.0005	7.48E-13	3.63E-07
cg24499975	1	MTR	TSS1500	4.54	0.0028	0.0004	8.38E-13	4.07E-07
cg24431486	11	CD81	Body	–1.39	–0.0023	0.0003	1.40E-12	6.79E-07

### Gene Expression by Quantitative Real-Time Polymerase Chain-Reaction

#### Validation of Methylation Study

**FIGURE 3:**
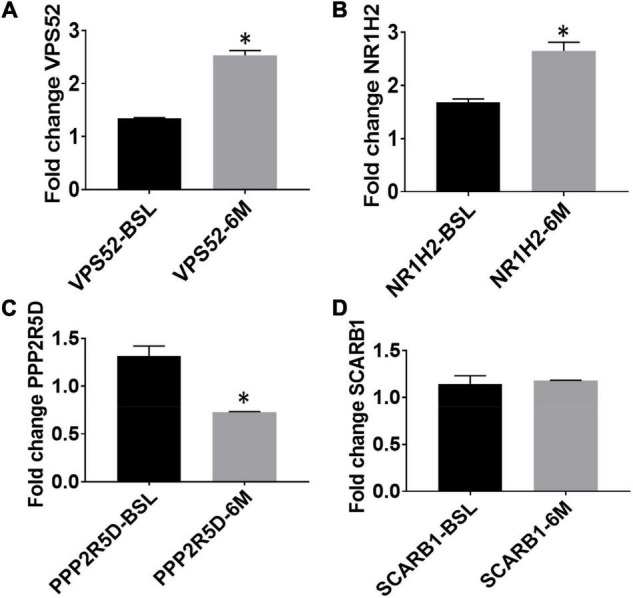
Expression levels of top exercise induced hypomethylated genes at baseline and following aerobic exercise intervention (**A**, VPS52; **B**, NR1H2; **C**, PPP2R5D; **D,** SCARB1). Gene expression by quantitative RT-PCR; mRNA levels of top 4 hypomethylated genes for VPS52; NR1H2; PPP2R5D; SCARB1 at baseline and post-exercise. Note: * Indicates statistically significant training-induced changes in gene expression levels.

We examined mRNA levels of the top 5 hypomethylated genes—*VPS52*; *NR1H2*; *PPP2R5D*; *SCARB1* and *ARTN*—at baseline and post-exercise, normalized to *GADPH* (housekeeping gene) levels. As expected, mRNA levels of *VPS52* and *NR1H2* increased significantly compared to baseline levels (Figures 3A,B). However, the level of *PPP2R5D* was significantly reduced following exercise intervention compared to the baseline (Figure 3C), and the 6-month expression level of *SCARB1* remained unchanged compared to baseline (Figure 3D). ARTN primers failed to amplify in the blood samples.

## Discussion

In this sample of elderly AA MCI study participants, a 6-month standardized, and supervised aerobic exercise-training influenced global DNA methylation changes. While the most significant hypomethylations involved *VSP52*, *SACRB1*, *ARTN*, *NR1H2*, and *PPPLR5D* genes (Table 2), these genes were not similarly influenced by training effects in the stretch exercise group. We discuss the biological plausibility of our most important findings, consistency with previous studies, and coherence with the known roles of exercise on our molecular findings, and therefore on the pathophysiology of age-associated cognitive decline.

It is now acknowledged that physical activity can enhance brain plasticity and improve cognition and wellbeing (Weinberg and Gould, 1999). Also, physical exercise can induce structural and functional changes in the brain and protect against neurodegeneration (Mandolesi et al., 2018). In a recent comprehensive review, Mandolesi et al. (2018) acknowledged the positive biological and psychological effects of physical activity on the brain through its enhancement of cognition, while Buchman et al. (2012) showed that physical activity reduced inflammation and improved memory in MCI and potentially Alzheimer’s Disease patients (Buchman et al., 2012). However, the mechanisms underlying this beneficial effect of exercise on memory are poorly understood. The present study is a logical extension of our previously published work on the effects of aerobic exercise on global array expression in AA MCI volunteers, showing that aerobic-exercise can down-regulate the expression of pro-inflammatory genes, concomitantly up-regulate anti-inflammatory genes, and promoted the expression of genes involved in axonal growth and neuronal survival (Iyalomhe et al., 2015). While the aerobic group underwent progressive exercise intensity and duration to achieve individualized 70% VO2 Max and endurance capacity, the stretch (control) group only undergone progressive stretch exercise. Overall, exercise training-induced increases in endurance capacity and reduced body weight in the aerobic exercise group affirmed training effects. Conversely, a slight decline in VO2max and unchanged body weight, in the stretch group, underscore less fitness effects. Collectively, this methylation study provides additional insights into the mechanism underlying the advantageous effect of exercise on memory.

While the biological mechanisms contributing to the health benefits of exercise are well-acknowledged, the exact mechanism underlying its effects on memory remains mostly unknown. Exercise can reshape the epigenome and induce significant changes in DNA methylation (Lindholm et al., 2014). Its ability to activate or silence specific genes may, therefore, explain its effects on health. For example, acute intensity exercise can alter the methylation of exercise-responsive genes, resulting in DNA hypomethylation in skeletal muscle (Barres et al., 2012). In contrast, a 6-month exercise program in previously sedentary middle-aged men increased methylation in adipose tissue (Rönn et al., 2013). For example, Rönn et al. (2013) provided a detailed map of the genome-wide DNA methylation pattern in human adipose tissue and linked exercise to altered adipose tissue DNA methylation. Therefore, these epigenetic changes can alter gene expression and metabolism and thus inform the advantageous effects of exercise on health (Ling and Ronn, 2014). Nonetheless, understanding the consequent biologic cascade resulting in long-lasting effects of regular exercise-training on DNA methylation needs a more nuanced understanding. For example, Soci et al. (2017) showed that exercise-induced changes in DNA methylation could alter several molecular mechanisms such as muscle contraction, increased mitochondrial mass, oxidative and non-oxidative mechanisms as well as cardiac and skeletal hypertrophy (Soci et al., 2017). Interestingly, some of these mechanisms have implications for fitness and cerebral hemodynamics.

The most significant effect of a 6-month exercise in our study is the hypomethylation of vacuolar protein sorting homolog 52 (*VPS52*) gene, supported by a corresponding increase in its mRNA levels. *VPS52* is a crucial part of the Golgi-associated retrograde complex that plays an important role in cellular inter-compartment transport and is highly expressed in the brain^1^. In one experiment, the cortex of Amyloid Precursor Protein (APP) knockout mice exhibited the upregulation of *VPS52* and a related protein. Conversely, lipid dysregulation and elevated cerebral APP are associated with decreased expression of *VPS52* (Castello, 2015). Therefore, given *VPS52* complex interaction with lipid regulation in the context of changing APP build-up, studies are needed to understand the role of *VPS52* on memory and human aging.

Hypomethylation near Nuclear Receptor Subfamily 1 Group H Member 2 (NR1H2) gene was also associated with exercise-associated increases in its mRNA expression. This observation is likely significant in that dysfunctionality of the NR1H2 gene can promote late-onset AD (Patel and Forman, 2004; Adighibe et al., 2016). Because dysregulated cholesterol levels can induce amyloidogenesis (Infante et al., 2010), NR1H2-mediated cellular efflux of cholesterol may protect against AD development.

Although exercise-training was associated with methylation changes in Scavenger receptor class B member 1 (SCARB1), Protein Phosphatase 2 Regulatory Subunit B’Delta (PPP2R5D), and Artemin (ARTN), corresponding changes in gene expression remained non-significant in our study. Therefore, further studies are needed to discern their biological roles. For instance, SCARB1 encodes high-density lipoprotein receptor that regulates cholesterol efflux from the peripheral tissue to the liver, is present in astrocytes and vascular smooth muscle cells in AD brain, and has been demonstrated to mediate adhesion of microglia to fibrillar amyloid-β (Aβ) (El Khoury et al., 1996; Thanopoulou et al., 2010; Jawaid and Khalil, 2011; Mulder et al., 2012). PPP2R5D is an interesting finding because its mutations can promote intellectual disability (Houge et al., 2015) and the development of spatially restricted tauopathy by deregulating CDK5 and GSK3-beta in mice lacking PPP2R5D subunits (Louis et al., 2011). The PPP2R5D gene encodes the protein B56-delta (B56δ), a part of (B subunit) of the phosphatase 2A (PP2A) enzyme, which dephosphorylates other specific proteins, to control their activation. Since most of the affected proteins are involved in active signaling, the activation/inhibition of PPP2R5D is often tightly controlled (Wang et al., 2008); thereby making its expression levels less informative. Further, ARTN may be critical to neuronal survival, given that protein signals through the RET receptor and GFR alpha three coreceptors encoded by ARTN support the survival of several peripheral neurons and a population of dopaminergic CNS neurons (Zihlmann et al., 2005).

However, we note that gene (ARTN) did not amplify in blood cells, and an alternative primer yielded a similar result. Also, we verified from GeneCards (Stelzer et al., 2016) that, while the ARTN gene is widely expressed in nervous tissues, it is not expressed in blood cells. Because phosphatases and kinases (e.g., PPP2R5D and GSK3β) are regulated more by post-translational modifications, increases in their activities are often associated with compensatory decreased mRNA levels through other pathways, and vice versa. While our methylation result is consistent with an expectation of increased levels of PPP2R5D, the incongruent decrease in mRNA levels suggests other regulatory factors may be involved. Also, our finding of training-induced changes in SCARB1 methylation is notable, given its expression in astrocytes (Mulder et al., 2012). Nonetheless, our findings are significant to current knowledge on exercise training-induced changes on blood methylation profile in MCI subjects.

The inaccessibility of living human brain tissue in AD studies motivated our consideration for using human peripheral blood to evaluate methylation profile following exercise intervention. Several studies have reported that DNA methylation patterns in human blood parallel epigenetic changes in the brain (Horvath et al., 2012; Walton et al., 2016). For example, Horvath et al. (2012) noted a correlation *r* = 0.9 across whole-blood and postmortem brain tissue methylation datasets, suggesting that blood DNA methylation profiles may be a promising surrogate for brain tissue (Horvath et al., 2012). Using blood obtained premortem, Davies et al. (2012) showed that individual methylation profile correlated with relevant postmortem brain tissue (cortex and cerebellum) methylation levels (correlation coefficient = 0.66–0.76) (Davies et al., 2012). Further, a recent systematic review of the DNA methylation studies showed that epigenetics changes in the peripheral blood of AD patients correlated with AD pathology (Wei et al., 2020). Acknowledged associations of DNA methylation in peripheral blood cells of AD patients with poor cognitive performances and APOE ε4 polymorphism are consistent with this report (Di Francesco et al., 2015). Thus, our approach to investigate methylation changes in peripheral blood following exercise intervention provides valuable insight into the mechanism underlying the effects of fitness adaptation on methylation, and potentially, on experimentally inaccessible human brain and neurodegeneration.

## Conclusion and Limitation

Our study revealed global changes in DNA methylations following 6 months of standardized and supervised aerobic exercise training in MCI subjects. The relevance of some of these genes to AD pathophysiology provides further evidence to the likely significance of aerobic exercise in moderating AD pathophysiology in MCI populations. An important limitation of this pilot study is the relatively small sample size. Nonetheless, we provide novel insight into the effect of aerobic exercise on the epigenome. Our plan includes testing these findings in larger samples and informing the genes’ relationship with cognitive phenotype and disease progression spectrum.

## Data Availability Statement

The datasets presented in this study can be found in online repositories. The names of the repository/repositories and accession number(s) can be found below: https://www.ebi.ac.uk/arrayexpress/ with accession E-MTAB-11166.

## Ethics Statement

The studies involving human participants were reviewed and approved by the Howard University Institutional Review Board (IRB). The patients/participants provided their written informed consent to participate in this study.

## Author Contributions

JN, EN, and TO conceived the study, cleaned, and analyzed the data. FB performed RNA isolation, cDNA preparation, conducted Q-PCR gene expression experimental setup, and analysis. JN, EN, FB, and TO wrote the manuscript. All authors contributed to study design and strategies for analyses, and participated in the review of the final manuscript.

## Conflict of Interest

The authors declare that the research was conducted in the absence of any commercial or financial relationships that could be construed as a potential conflict of interest.

## Publisher’s Note

All claims expressed in this article are solely those of the authors and do not necessarily represent those of their affiliated organizations, or those of the publisher, the editors and the reviewers. Any product that may be evaluated in this article, or claim that may be made by its manufacturer, is not guaranteed or endorsed by the publisher.

## Footnotes

1
https://www.ncbi.nlm.nih.gov/gene/6293

## Abbreviations

AA: African American
AD: Alzheimer’s disease
CpG: CG dinucleotide
FDR: false discovery rate
GEMS: gene, exercise, and memory study
GO: gene ontology
MCI: mild cognitively impaired
RT-PCR: reverse transcription polymerase chain reaction
VO2max: maximal oxygen consumption.
