# Supplementary material for: Aerobic Exercise Training-Induced Changes on DNA Methylation in Mild Cognitively Impaired Elderly African Americans: Gene, Exercise, and Memory Study - GEMS-I
Source: Front Mol Neurosci. 2022 Jan 17;14:752403. doi: 10.3389/fnmol.2021.752403 (PMC8802631; doi:10.3389/fnmol.2021.752403)
Supplement: Supplementary file 1 [file Table_1.pdf]

Supplemental Table 1: Linear Mixed Effects Analysis to Examine the Effects of Stretch Exercise on the Top 10 CpG Sites Identified in Aerobic Exercise Group.

Top 10 Aerobic CpGs			Aerobic (N = 11)			Stretch (N = 8)		
CpG	CHR	Gene Symbol	beta	se	p	beta	se	p
cg00160018	6	VPS52	-0.0020	0.0002	5.38E-26	-0.006	0.013	0.671
cg11198639	12	SCARB1	-0.0063	0.0006	8.83E-25	0.023	0.017	0.189
cg17414508	1	ARTN	-0.0049	0.0005	6.10E-25	0.005	0.011	0.651
cg24440997	19	NR1H2	-0.0029	0.0003	2.06E-18	0.007	0.014	0.642
cg14385961	6	PPP2R5D	-0.0007	0.0001	9.83E-18	0.016	0.011	0.172
cg11132661	12		-0.0018	0.0002	2.01E-17	-0.001	0.010	0.913
cg02170785	14		-0.0044	0.0006	9.71E-16	0.010	0.017	0.573
cg02469461	2	CAB39	-0.0015	0.0002	1.42E-14	-0.008	0.019	0.690
cg22988430	15	SNORD115-41	-0.0052	0.0007	1.84E-14	0.006	0.019	0.765
cg08732418	15	DLL4	-0.0061	0.0008	2.13E-14	0.001	0.014	0.954
